# Anti-Atherosclerotic Effects of a Phytoestrogen-Rich Herbal Preparation in Postmenopausal Women

**DOI:** 10.3390/ijms17081318

**Published:** 2016-08-11

**Authors:** Veronika A. Myasoedova, Tatyana V. Kirichenko, Alexandra A. Melnichenko, Varvara A. Orekhova, Alessio Ravani, Paolo Poggio, Igor A. Sobenin, Yuri V. Bobryshev, Alexander N. Orekhov

**Affiliations:** 1 Centro Cardiologico Monzino, IRCCS, Milan I-20138, Italy; veronika.myasoedova@gmail.com (V.A.M.); alessio.ravani@ccfm.it (A.R.); paolo.poggio@ccfm.it (P.P.); 2 Institute of General Pathology and Pathophysiology, Moscow 125315, Russia; zavod@ifarm.ru (A.A.M.); sobenin@cardio.ru (I.A.S.); a.h.opexob@gmail.com (A.N.O.); 3 Institute for Atherosclerosis Research, Skolkovo Innovative Center, Moscow 143025, Russia; t-gorchakova@mail.ru (T.V.G.); varvaraao@gmail.com (V.A.O.); 4 Russian Cardiology Research and Production Complex, Moscow 121552, Russia; 5 Faculty of Medicine, School of Medical Sciences, University of New South Wales, Sydney, NSW 2052, Australia; 6 School of Medicine, University of Western Sydney, Campbelltown, NSW 2560, Australia; 7 Department of Biophysics, Biological Faculty, Moscow State University, Moscow 119991, Russia

**Keywords:** atherosclerosis, menopause, herbal preparation, prevention, intimal medial thickens, isoflavonoids, phytoestrogens

## Abstract

The risk of cardiovascular disease and atherosclerosis progression is significantly increased after menopause, probably due to the decrease of estrogen levels. The use of hormone replacement therapy (HRT) for prevention of cardiovascular disease in older postmenopausal failed to meet expectations. Phytoestrogens may induce some improvements in climacteric symptoms, but their effect on the progression of atherosclerosis remains unclear. The reduction of cholesterol accumulation at the cellular level should lead to inhibition of the atherosclerotic process in the arterial wall. The inhibition of intracellular lipid deposition with isoflavonoids was suggested as the effective way for the prevention of plaque formation in the arterial wall. The aim of this double-blind, placebo-controlled clinical study was to investigate the effect of an isoflavonoid-rich herbal preparation on atherosclerosis progression in postmenopausal women free of overt cardiovascular disease. One hundred fifty-seven healthy postmenopausal women (age 65 ± 6) were randomized to a 500 mg isoflavonoid-rich herbal preparation containing tannins from grape seeds, green tea leaves, hop cone powder, and garlic powder, or placebo. Conventional cardiovascular risk factors and intima-media thickness of common carotid arteries (cIMT) were evaluated at the baseline and after 12 months of treatment. After 12-months follow-up, total cholesterol decreased by 6.3% in isoflavonoid-rich herbal preparation recipients (*p* = 0.011) and by 5.2% in placebo recipients (*p* = 0.020); low density lipoprotein (LDL) cholesterol decreased by 7.6% in isoflavonoid-rich herbal preparation recipients (*p* = 0.040) and by 5.2% in placebo recipients (non-significant, NS); high density lipoprotein (HDL) cholesterol decreased by 3.4% in isoflavonoid-rich herbal preparation recipients (NS) and by 4.5% in placebo recipients (*p* = 0.038); triglycerides decreased by 6.0% in isoflavonoid-rich herbal preparation recipients (NS) and by 7.1% in placebo recipients (NS). The differences between lipid changes in the isoflavonoid-rich herbal preparation and placebo recipients did not reach statistical significance (*p* > 0.05). Nevertheless, the mean cIMT progression was significantly lower in isoflavonoid-rich herbal preparation recipients as compared to the placebo group (6 μm, or <1%, versus 100 μm, or 13%; *p* < 0.001 for the difference). The growth of existing atherosclerotic plaques in isoflavonoid-rich herbal preparation recipients was inhibited by 1.5-fold (27% versus 41% in the placebo group). The obtained results demonstrate that the use of isoflavonoid-rich herbal preparation in postmenopausal women may suppress the formation of new atherosclerotic lesions and reduce the progression of existing ones, thus promising new drug for anti-atherosclerotic therapy. Nevertheless, further studies are required to confirm these findings.

## 1. Introduction

Postmenopausal status increases cardiovascular risk due to accelerated atherosclerosis progression. Cardiovascular diseases remain the leading cause of mortality and morbidity among postmenopausal women. The cardiovascular risk related to postmenopausal status is predominately due to the rapid decrease of estrogen levels, which are attributed to the indirect protective effect on lipid and glycemic control, and to the direct effect on endothelial function [1,2]. The use of hormone replacement therapy (HRT) in cardiovascular prevention failed to meet expectations and it has been recognized that long-term use of hormone therapy may actually increase the risk of cardiovascular disease (CVD) in postmenopausal women, as shown in the Heart and Estrogen/progestin Replacement Study (HERS) trial, which was conducted in older postmenopausal women with established coronary heart disease (CHD) [3,4]. The known side effect of HRT—that is, an increased risk of malignant hormone-dependent tumors—also produced a negative impact on the perspectives of such therapy [5,6]. The general outcome of several large-scale trials was that neither estrogen nor estrogen/progestin decreased cardiovascular disease [7]. However, later analysis has shown that HRT started in early postmenopause provides cardiovascular benefit and no harm [8,9]. In spite of these findings, the expert opinion says that HRT should not be used for the primary or secondary prevention of CHD; it should be limited to the treatment of menopausal symptoms at the lowest effective dosage over the shortest duration possible, and continued use should be re-evaluated on a periodic basis [10,11].

Phytoestrogens, mainly isoflavonoids, are believed to be an alternative to HRT in postmenopausal women. Phytoestrogens comprise a rather heterogeneous group of natural compounds of plant origin with structures similar to estrogen E2. Three of the most active compounds are coumestans, prenylflavonoids, and isoflavones. The hypothetical effects of phytoestrogens are mediated via estrogen receptors (ERα and ERβ), and the G protein-coupled estrogen receptor (GPER). It is also known that phytoestrogens have high affinity for ERβ, which explains their different action from endogenous estrogens [12]. Similar to endogenous estrogens, phytoestrogens may provide beneficial effects on cardiovascular system through the effects on the vascular endothelium [13], vascular smooth muscle cells [14,15], intracellular cholesterol metabolism [16,17,18], extracellular matrix synthesis [19], and vascular inflammation [20].

Dietary supplementation with phytoestrogens may inhibit the development of atherosclerotic lesions. It has been demonstrated that phytoestrogens from grapes prevent cholesterol accumulation in blood-derived cultured monocytes from postmenopausal women [17]. Animal studies support the anti-atherogenic properties of phytoestrogens; for example, genistein inhibited atherogenesis in hypercholesterolemic rabbits mostly via its beneficial effects on endothelial dysfunction [21]. Resveratrol (stilbene with known estrogen-like activity) exhibited multiple anti-atherogenic effects [22], including inhibition of intimal hyperplasia [23] and inhibition of low density lipoprotein (LDL) oxidation [24]. The results of experimental studies demonstrate that phytoestrogens have a potential in anti-atherosclerotic therapy, because they are able to modulate several mechanisms of atherosclerosis progression.

Previously, in an ex vivo model, we evaluated the anti-atherosclerotic effect of phytoestrogen-rich plants and their combinations [17]. Based on the results of dose titration studies, qualitative compositions of isoflavonoid-rich anti-atherosclerotic herbal preparation—active ingredients: tannins from grape seeds, green tea leaves, hop cone powder, and garlic powder—was developed. The aim of the present study was to investigate the effect of this isoflavonoid-rich herbal preparation on the progression of subclinical carotid atherosclerosis in healthy postmenopausal women.

The intima-media thickness of common carotid arteries (cIMT) measured by B-mode ultrasound is a widely accepted marker of subclinical atherosclerosis; it is well correlated with the degree of coronary atherosclerosis and is a significant predictor of clinical manifestations of atherosclerosis. This instrumental marker is used in clinical and epidemiological studies to assess the impact of conventional and novel cardiovascular risk factors and treatment regimens on atherosclerosis progression [25,26,27]. Several clinical trials were aimed at the assessment of the effects of HRT or phytoestrogens on cIMT progression in postmenopausal women [28,29,30,31]. Thus, in this study we have used ultrasound examination of common carotid arteries and cIMT measurement as a tool for quantitative assessment of atherosclerosis, with annual cIMT progression as the endpoint.

## 2. Results

### 2.1. Baseline Data

In total, 157 asymptomatic postmenopausal women were included in the study, 77 in the isoflavonoid-rich herbal preparation group, and 80 in the placebo group. The groups did not differ in age, body mass index, smoking status, family history of coronary artery disease, blood level of triglycerides, and high density lipoprotein (HDL) cholesterol (HDL-C), the prognostic risk of myocardial infarction, and sudden death. No difference was found between groups in mean and maximum cIMT, and in the size of asymptomatic carotid atherosclerotic plaques. However, systolic and diastolic blood pressure levels were significantly higher in placebo group, whereas total cholesterol and low density lipoprotein (LDL) cholesterol (LDL-C) levels were significantly higher in isoflavonoid-rich herbal preparation recipients at the baseline. The proportion of patients with diabetes was also higher in isoflavonoid-rich herbal preparation recipients. Baseline characteristics of study participants are given in Table 1.

### 2.2. Follow-up

Of the 157 enrolled study participants, 131 completed study protocol (57 isoflavonoid-rich herbal preparation recipients and 74 placebo recipients). Among dropouts, 16 were lost for follow-up examination (12 in the isoflavonoid-rich herbal preparation recipients group, 4 in the placebo group) and 10 refused to visit for personal reasons and withdrew their informed consent (8 in the isoflavonoid-rich herbal preparation recipients group, 2 in the placebo group). In participants available for follow-up examination no adverse or side effects were registered in both groups. Thus, the higher dropout rate observed in isoflavonoid-rich herbal preparation recipients can hardly be explained by some adverse or side effects of the study medication. The comparison of odds ratios for dropout have shown that the observed dropout values are better explained by chance, taking into account rather small sample size.

After 12-month follow-up, blood pressure, lipid profile, as well as ultrasound characteristics of carotid atherosclerosis were determined in both groups. Blood lipid levels decreased in both groups, and in the placebo group these changes were statistically significant for total cholesterol (from 252 to 239 mg/dL, or by 5.2% reduction, *p* = 0.020) and HDL-C (from 74 to 71 mg/dL, or by 4.5% reduction), in isoflavonoid-rich herbal preparation recipients for total cholesterol (from 271 to 254 mg/dL, or by 6.3% reduction) and LDL-C (from 170 to 157 mg/dL, or by 7.6% reduction; *p* = 0.040). The decrease in serum triglyceride levels was statistically insignificant in both groups. The difference between lipid changes in isoflavonoid-rich herbal preparation and placebo recipients did not reach statistical significance neither for total cholesterol, nor for LDL-C, HDL-C, and triglycerides. Blood pressure levels and body mass index did not change in either group. The changes of clinical and biochemical characteristics from the baseline after 12-month follow-up are given in Table 2.

**Figure 1:**
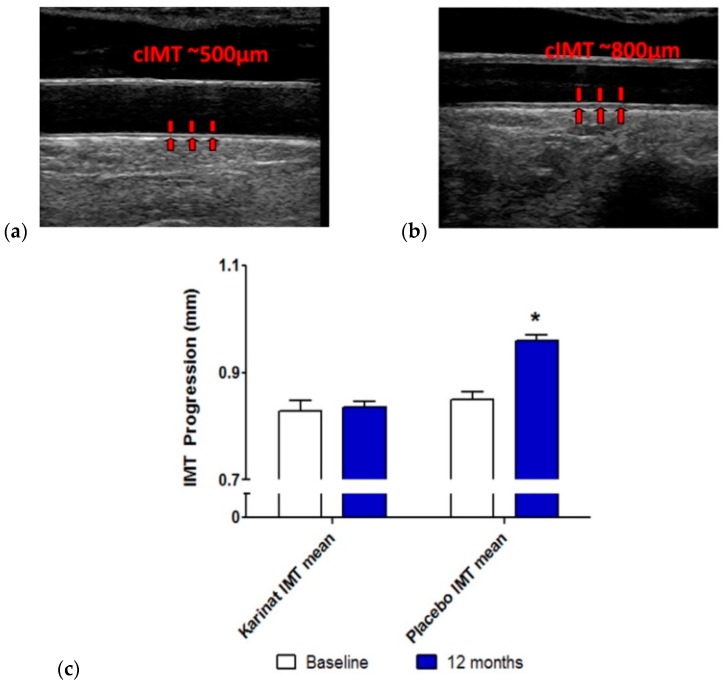
Actual individual ultrasound images and cIMT values at the baseline and after 12-month follow-up. (**a**) Normal cIMT in apparently healthy postmenopausal women; (**b**) Abnormally increased cIMT in apparently healthy postmenopausal women; (**c**) Dynamics of cIMT in isoflavonoid-rich herbal preparation and placebo recipients at the baseline (open bars) and after 12-months follow-up (filled bars). The data are presented as mean and S.E.M. *, represents a significant difference between baseline and follow-up cIMT values; *p* < 0.05.

In isoflavonoid-rich herbal preparation recipients, no significant increase of mean cIMT was observed; the increment accounted for 6 μm (less than 1%), and the growth of atherosclerotic plaque growing accounted for 27% of the baseline value. C, in the placebo group the rate of atherosclerosis progression was higher (i.e., the increment of mean cIMT accounted for more than 100 μm (13%) and the growth of atherosclerotic plaques accounted for 40% of the baseline value) (Table 3). There was a significant difference between the isoflavonoid-rich herbal preparation and placebo recipients in mean cIMT increase over 12-month follow-up (*p* < 0.001), but not in maximum cIMT increase (*p* = 0.89) or in the growth of existing atherosclerotic plaques (*p* = 0.30). The samples of actual individual ultrasound images and the mean values of cIMT at the baseline and after follow-up are shown in Figure 1.

## 3. Discussion

The results of this study have demonstrated that mean cIMT progression was slower in asymptomatic postmenopausal women who received isoflavonoid-rich herbal preparation, as compared to women who received the placebo. In addition, the herbal preparation decreased the total cholesterol, LDL-C levels, and suppressed cIMT progression after 12 months of herbal preparation administration. It should be noted that in our study, the baseline LDL-C and total cholesterol level, as well as the prevalence of diabetes in isoflavonoid-rich herbal preparation recipients were higher than in the placebo group; these risk factors of atherosclerosis could suggest more pronounced cIMT and plaque progression. However, we have seen the reverse effect; therefore, it may be expected that the anti-atherosclerotic potency of isoflavonoid-rich herbal preparation may even be underestimated in this study. On the other hand, the reduction in total cholesterol and LDL-C after 12-month follow-up in the isoflavonoid group could be due to regression towards the mean, since they were higher at the baseline.

CVD related to atherosclerotic process is responsible for the majority of deaths in postmenopausal women. The prevention of the lipid accumulation in cells is the key mechanistic factor for inhibition of atherosclerotic plaque formation at the early stages of atherosclerosis progression. Phytoestrogens have the capacity to affect plasma lipid profile, but little is known regarding their effects on atherosclerosis progression. In women undergoing coronary angiography for suspected myocardial ischemia, beneficial association between blood levels of the phytoestrogen daidzein and lipoproteins, particularly lower triglycerides and higher HDL-C levels were previously reported [32]. The main association of phytoestrogens with lipoprotein levels was incrementally related to diadzein, but not with other lipoprotein modulators. In another clinical study it has been shown that isoflavones induce the reduction of total cholesterol and LDL-C plasma levels without affecting triglycerides or HDL-C levels [33]. Our results are in line with previous findings; however, in our study blood lipid levels were decreased in both groups: in the placebo group, these changes were significant for total cholesterol and HDL-C; and in the isoflavonoid-rich herbal preparation recipients, for total cholesterol and LDL-C. The ability of phytoestrogens to reduce the accumulation of cholesterol in cells is a possible mechanism to explain the effects on mean cIMT. Previously, we have evaluated the anti-atherogenic effect of phytoestrogen-rich plants using an ex vivo model based on primary cultures of monocytes isolated from the blood of healthy donors. In this model, the ability of human serum to induce accumulation of cholesterol in cultured cells (serum atherogenicity) was measured, as well as the effect of single dose oral administration of plant extract on serum atherogenicity [17,34]. Grape seeds extract (100 mg) lowered serum atherogenicity by 71%, 78%, and 81% at 2, 4, and 6 h after oral intake of a single dose. Similar effects were observed for hop cones (250 mg), garlic powder (150 mg), sage leaves (100 mg), green tea leaves (250 mg), sea kelp (500 mg), fucus (250 mg), and carrot (1000 mg). The ability of soya beans extract (35 mg) to lower serum atherogenicity by 28%, 38%, and 30% at 2, 4, and 6 h after a single dose administration, respectively, was demonstrated [17,35].

The main endpoint of the current study was to identify the annual changes in cIMT progression. Several studies demonstrated that cIMT is a significant and independent predictor of cardiovascular events, and allows for non-invasive evaluation of early atherosclerosis progression in asymptomatic patients [36]. Only a few clinical trials investigated the effect of phytoestrogens on atherosclerosis progression in postmenopausal women. In the recent long-term intervention trial (2.7 years) with soy isoflavones, the inhibition of subclinical atherosclerosis progression evaluated by cIMT was demonstrated. Healthy postmenopausal women were randomized in two groups; the first group who received daily supplement with 25 g soy protein containing 91 mg of isoflavones, and the second group who received a placebo. In both groups, the increment of cIMT was observed. However, in the soy group the cIMT progression was not statistically significant (*p* = 0.35), and was 16% lower than in the placebo group. That study has enrolled 350 participants, and the duration was more than two years. The authors suggested that further use of isoflavone-rich dietary supplements would allow achieving the significant difference in the rate of atherosclerosis progression between the two groups [28].

In our study, mean cIMT changes in both groups were observed. However, in herbal preparation recipients this increase was negligible, but in the placebo group the increment was significantly higher than in herbal preparation recipients, and accounted for 111 μm, or 13% increase. This fact indicates that in postmenopausal women the rate of carotid atherosclerosis progression is notably high. In the study by Rossi et al. [37], the mean cIMT progression accounted for 103 μm (range from −250 to 567 μm; IQR from 0 to 200 μm) per year in hypertensive postmenopausal women, and this progression rate is very close to our data. It should be noted that in our study the difference between the two groups in systolic and diastolic blood pressure at the baseline was statistically significant (135/83 versus 127/79 in placebo group and herbal preparation group, respectively), and this fact may partly explain the rather high progression of cIMT in the placebo group, which has not been replicated in any other studies [29,30]. On the other hand, Colacurci et al. have demonstrated rather similar cIMT progression rates in non-hypertensive women [31]. The limitations of our study, such as the duration of the follow-up and rather limited sample size, did not allow defining the proportion of cIMT progression rates in placebo recipients explained by the higher blood pressure.

It should be noted that the time since menopause is of the essence when studying atherosclerosis progression and medical intervention. There exists an opinion that timely HRT may offer protection against CVD, whereas in older women there may be cardiovascular harm associated with HRT use [8,9]. Hodis et al. have recently demonstrated that anti-atherosclerotic effects of HRT on cIMT progression differed between early and late postmenopause. Oral estradiol therapy was associated with less progression of subclinical atherosclerosis measured as cIMT dynamics than was placebo when therapy was initiated within six years after menopause, but not when it was initiated ten or more years after menopause [29]. Thus, estradiol was shown to be effective in reducing cIMT progression. On the other hand, in the Kronos Early Estrogen Prevention Study (KEEPS) performed in more than 700 healthy women aged 42 to 59 within three years after menopause, the carotid ultrasound studies showed similar rates of progression of cIMT in all three treatment groups (0.45 mg a day of Premarin—an oral conjugated equine estrogen (o-CEE)—or 50 µg a day of transdermal estradiol via a Climara patch, or placebo) over the four years of study. However, these changes were reported to be generally small; therefore, slow cIMT progression limited the statistical power to detect any differences among the groups [30]. According to the results of our study, isoflavonoid-rich herbal preparations may provide a direct anti-atherosclerotic effects, but no direct comparisons with estrogens were performed. In general, it may be proposed that anti-atherosclerotic action of drugs should be realized via prevention of intracellular cholesterol accumulation in vascular wall cells, but it is unclear if estrogens may possess the same mechanistic effect at the cellular level. In our previous studies on prevention of intracellular cholesterol accumulation, modified LDL were used to induce intracellular lipid deposition, and the effects of drugs or chemical compounds mainly related to LDL binding, uptake, internalization, and metabolism in cells were in focus. In contrast, Wang et al. explored the alternative way of preventing foam cell formation via cholesterol efflux modulation. They have demonstrated that 17β-estradiol promotes cholesterol efflux from vascular smooth muscle cells and reduces foam cell formation via ERβ- and liver X receptor (LXR) α-dependent upregulation of ABCA1 and ABCG1 [38]. Another mechanism of atherosclerosis prevention may be related to anti-inflammatory effects, and it was shown that estradiol can regulate monocyte chemotactic protein-1 (MCP-1) in human coronary artery smooth muscle cells [39], increase prostacyclin synthesis in cells from atherosclerotic lesions [40], impair endothelial function in postmenopausal women [41], transform growth factor activity [42], and attenuate atherogenesis via selective estrogen receptor beta modulator 8β-VE2 [43]. On the other hand, anti-inflammatory effects of phytoestrogens are also known [44,45,46,47]. Therefore, anti-atherogenic effects of both estrogens and isoflavonoids are not limited to the inhibition of direct accumulation of cholesterol in cells only.

Finally, the findings of our study are in line with the results obtained from the study aimed to evaluate the effect of selective estrogen receptor modulator Raloxifene on atherosclerosis progression in postmenopausal clinically healthy women. In a prospective study enrolling 155 postmenopausal women without clinical manifestations of CVD, study participants were randomized in two groups, receiving Raloxifene 60 mg daily or placebo for 18 months. The cIMT progression for 18 months was 11.2 μm in Raloxifene group versus 85.7 μm in the placebo group (*p* < 0.004). Thus, the lower risk of cIMT progression was demonstrated in Raloxifene recipients (odds ratio = 0.41; 0.32–0.70 at a 95% confidence interval) [31].

Nevertheless, our study has certain limitations. The main one is the duration of the follow-up, only 12 months. Long-term effects of isoflavonoid-rich dietary supplement Karinat need to be further studied in order to evaluate its effects on main cardiovascular risk factors and long-term outcomes, such as myocardial infarction and stroke [48]. Indeed, longer observation may help to better understand the effects of isoflavonoid-rich herbal preparations on main outcomes of CVD, but in this study that was not the primary endpoint. The second notable limitation is a rather small sample size. To interpret the results from our study, the limited number of enrolled subjects needs to be taken into consideration, as it may lead to confounding results, despite randomization. Finally, it should be noted that the effect of isoflavonoids or other estrogen-like molecules on cardiovascular health may be realized more through endothelial function/dysfunction. In our study we have evaluated only the effects on lipids, and the effect of treatment on the arterial wall that reflects atherosclerotic profile. It should be expedient to study the effects of isoflavonoid-rich herbal medications also on endothelial function using, for example, flow-mediated dilatation.

## 4. Materials and Methods

### 4.1. Study Medication

Isoflavonoid-rich herbal preparation contained tannins from grape seeds (*Vitis vinifera* L.), green tea leaves (*Camellia sinensis* L.), hop cone powder (*Hunulus lupulus*), and garlic powder (*Allium sativum* L.). Commercially available purified compounds were used. This preparation was officially registered as a dietary supplement “Karinat” and was manufactured by INAT-Farma (Moscow, Russia). The quantified chemical constituents are provided in Table 4. The content of toxic elements, pesticides, dichlorodiphenyltrichloroethane (DDT), and its metabolites and microbiological purity have been controlled. The measurement of cathechines and allicin contents was performed by high performance liquid chromatography (HPLC). Based on previous dose titration studies, the dosage regimen for isoflavonoid-rich herbal preparation was determined [17]. The quantity of herbal constituents was 500 mg per capsule; a total of three capsules were given daily, independently of meals, for 12 months. The dosage regimen of Karinat (three capsules daily) provides for estimated daily intake of 27.3 mg procyanidin, 2.5 mg genistein, 11.8 mg daidzein, 4.6 mg flavones, 3.5 mg resveratrol, and 44.6 mg of other polyphenolic compounds [49].

### 4.2. Study Design

The study was performed in the Outpatient Clinic Nº 202 at Moscow State University. In total, 157 asymptomatic postmenopausal women were included in double-blind, placebo-controlled clinical study (ClinicalTrials.gov Identifier, NCT01742000). The inclusion criteria were as follows: the menopausal state (physiological or surgical) at least for the last five years; maximum cIMT more than 0.80 mm as determined by ultrasound B-mode examination of carotid arteries; the absence of climacteric syndrome (no more than two points by the Blatt-Kupperman score [50]). Exclusion criteria were as follows: the use of HRT during the peri- and postmenopausal period; the use of the lipid-lowering drugs for at least six months prior to inclusion; the absence of signed informed consent; the permanent use of sugar-lowering drugs (more than two months per year); the history of myocardial infarction, stroke, heart failure, uncontrolled hypertension (blood pressure above 145/95 mm·Hg in patients receiving antihypertensive treatment); cancer; chronic kidney disease; chronic liver disease; intolerability of the components of isoflavonoid-rich herbal preparation; and/or adverse reactions and/or side effects revealed during the follow-up. Some inclusion and exclusion criteria were intentionally defined to be compatible in general with those used in the KEEPS [51], in order to allow the possibility of tentative comparison of the rate of cIMT progression in early menopausal women. The use of lipid- and sugar-lowering medications was considered as a limitation for the inclusion in the study, since they may provide their own effects on cIMT progression [52,53,54,55]. The study participants were randomized into two groups: the first group who received isoflavonoid-rich herbal preparation (Karinat, INAT-Farma), three capsules daily for 12 months, and the second group who received a placebo. Karinat and placebo capsules looked identical.

### 4.3. Baseline Examination

Clinical and laboratory examinations were performed at the inclusion to the study and included anthropometric parameters (i.e., age, body mass index, blood pressure); personal and family history of arterial hypertension, diabetes mellitus, and coronary heart disease; lipid profile (i.e., cholesterol, triglycerides, LDL-C, HDL-C), B-mode ultrasound examination of common carotid arteries, as well as evaluation of the severity of menopausal symptoms by the Blatt-Kupperman score [50].

### 4.4. Follow-up Examination

Follow-up examination was performed after 12 months of treatment and included the same clinical and laboratory examinations, as at the baseline. The rate of cIMT progression was the primary endpoint of the study, since it is conventionally used as an intermediate outcome for vascular risk estimation. It was demonstrated that cIMT progression may be rather slow [56]. We have investigated the cIMT progression in healthy postmenopausal women after five years of menopause, and in this age the cIMT progression was expected to be accelerated. Therefore, 12-month follow-up was considered to be sufficient to detect significant changes in cIMT in this cohort. On the other hand, the studies aimed to investigate the atherosclerosis progression and/or the role of anti-atherosclerotic therapy in postmenopausal women employed a one year (12-month) follow-up [37,57]. These considerations prevented us from evaluating lipid results and carotid arteries earlier than 12-month intervals.

### 4.5. Blood Sampling and Lipid Measurements

Venous blood was taken after overnight fasting. Commercially available enzymatic kits (Fluitest CHOL, Fluitest TG, Fluitest HDL-CHOL, Analyticon, Potsdam, Germany) were used for total cholesterol, triglycerides, and HDL-C measurements in blood serum. LDL-C was calculated with the Friedewald formula.

### 4.6. Calculation of Prognostic Cardiovascular Risk

The calculation of ten-year prognostic risk of fatal and non-fatal myocardial infarction and sudden death was performed in accordance with PROCAM Study-derived Cox proportional hazards model [58]. Such variables as female gender, age, blood pressure, smoking, diabetes mellitus, total cholesterol, triglycerides, and family history of acute myocardial infarction (first-grade relatives with the events occurred before the age of 60 years), were used for risk calculation, and the regional adjustment coefficient was applied [59].

### 4.7. Carotid Artery Ultrasound Examination

To examine the carotid arterial wall, B-mode high-resolution ultrasonography with a linear vascular 7.5 MHz probe (SSI-1000 scanner, SonoScape, Shenzhen, China) was performed by three operators. The examination included a scanning of the left and right common carotid arteries, the carotid sinus area, as well as external and internal carotid arteries, with a focus on the far wall of the artery in three fixed projections (anterior, lateral, and posterior [60]). The measurements were performed on distal 10 mm of common carotid artery (the opposite site from carotid sinus of the common carotid artery). Reproducibility of cIMT measurements was assessed according to the protocol of the IMPROVE Study [61]. Within-operator coefficient of variation (CV) was 2.6%; reproducibility coefficient accounted for 0.040. The frozen scans were digitized for subsequent cIMT quantitative measurement using specialized software package (M’Ath ver. 3.1, IMT, Paris, France). The cIMT far wall was measured as the distance from the leading edge of the first echogenic zone to the leading edge of the second echogenic zone. The measurements were performed by an independent certified reader in a blinded manner. The mean of all measurements in the anterior, posterior, and lateral projections were considered as integral measurements of cIMT.

### 4.8. Statistical Analysis

The significance of differences was analyzed with the IBM SPSS 21.0 program package (IBM, Armonk, NY, USA). The Mann–Whitney statistics or *t*-test were applied for between-group valuations, Wilcoxon statistics were performed for within-group effect comparisons, and Pearson's chi-squared was used for the assessment of nominal variables distributions. Pearson's correlation analysis and regression analysis were applied for the evaluation of the relationship between the values of risk changes and clinical and biochemical variables. The data are reported as the mean and standard deviation (SD). The differences were considered statistically significant at the 0.95 level of confidence (*p* < 0.05).

## 5. Conclusions

Our data suggest that the use of the isoflavonoid-rich herbal preparation Karinat may play an important role in the prevention of atherosclerosis progression in postmenopausal women, since it essentially suppressed the formation of new atherosclerotic lesions approximately by 1.5-fold and slowed the progression of existing ones. Further evaluation of the study results should be based on the precise knowledge of cardioprotective, metabolic, and anti-atherosclerotic effects of isoflavonoids, other phytoestrogens and their combinations. The isoflavonoid-rich herbal preparation used in our study provides intake of a mix of polyphenolic compounds, including procyanidin, genistein, daidzein, flavones, and resveratrol [49], but the role of each compound in the inhibition of cIMT and plaque progression remains to be unraveled. Our study unambiguously suggests that there is the potential for this herbal supplement for the prevention of atherosclerosis in postmenopausal women. However, it is worth noting that the present study is preliminary in nature, and the herbal preparations are still limited to prevention, but not treatment.

Thus, the use of isoflavonoid-rich herbal preparations may be considered nowadays as a promising approach for the development of anti-atherosclerotic therapy. Nevertheless, further studies are required to confirm this possibility.

## Figures and Tables

**Table 1:** Baseline characteristics of study participants.

Variable	Isoflavonoid-Rich Herbal Preparation Recipients, *n* = 77	Placebo Recipients, *n* = 80	*p*-Value
Age, years	65 (7)	65 (6)	0.804
Body mass index, kg/m^2^	27.1 (4.0)	26.9 (3.8)	0.782
Systolic BP, mm·Hg	127 (13)	135 (18)	0.006
Diastolic BP, mm·Hg	79 (8)	83 (9)	0.006
Smoking, *n* (%)	3 (5)	7 (10)	0.362
Diabetes, *n* (%)	6 (11)	1 (1)	0.022
Hypertension, *n* (%)	29 (51)	41 (56)	0.532
Family history of CAD, *n* (%)	16 (30)	19 (26)	0.634
Family history of hypertension, *n* (%)	30 (53)	37 (51)	0.827
Family history of diabetes, *n* (%)	5 (9)	9 (14)	0.387
Total cholesterol, mg/dL	271(55)	252 (42)	0.024
Triglycerides, mg/dL	134 (78)	126 (51)	0.456
HDL-C, mg/dL	74 (15)	74 (18)	0.745
LDL-C, mg/dL	170 (47)	153 (42)	0.034
Risk of MI, PROCAM score, %	1.64 (3.34)	1.24 (1.40)	0.363
cIMT mean, mm	0.829 (0.138)	0.849 (0.133)	0.415
cIMT max, mm	0.950 (0.172)	0.981 (0.161)	0.287
Carotid plaque, relative size, score	0.77 (0.78)	0.76 (0.72)	0.908

The data are presented as mean and standard deviation (in parentheses), if not otherwise indicated. BP: blood pressure; cIMT: intima-media thickness of common carotid arteries; HDL-C: high density lipoprotein cholesterol; LDL-C: low density lipoprotein cholesterol; MI: myocardial infarction; *n*: number of cases.

**Table 2:** The changes of characteristics of study participants after 12-month follow-up.

Variable	Isoflavonoid-Rich Herbal Preparation Recipients, *n* = 56		Placebo Recipients, *n* = 71	
	Change	*p*-Value	Change	*p*-Value
Body mass index, kg/m^2^	−0.01 (0.8)	0.978	−0.07 (1.6)	0.708
Systolic BP, mm·Hg	5 (19)	0.051	−1 (18)	0.666
Diastolic BP, mm·Hg	−1 (8)	0.806	−1 (9)	0.150
Total cholesterol, mg/dL	−17 (46)	0.011	−13 (41)	0.020
Triglycerides, mg/dL	−9 (53)	0.232	−9 (40)	0.106
HDL-C, mg/dL	−3 (11)	0.114	−3 (12)	0.038
LDL-C, mg/dL	−13 (45)	0.040	−8 (39)	0.126

The data are presented as mean and standard deviation (in parentheses).

**Table 3:** Carotid atherosclerosis progression.

Variable	Isoflavonoid-Rich Herbal Preparation Recipients, *n* = 56		Placebo Recipients, *n* = 71	
	Change	*p*-Value	Change	*p*-Value
cIMT mean, μm	+6 (85)	0.6	+111 (91)	<0.001
cIMT max, μm	+8 (101)	0.6	+4 (220)	0.9
Carotid plaque, score	+0.21 (0.59)	0.009	+0.31 (0.55)	<0.001

The data are presented as mean and standard deviation (in parentheses).

**Table 4:** Herbal and technological composition of isoflavonoid-rich herbal preparation “Karinat”.

Constituent	Mg Per Capsule	%
*Humulus lupulus* L*.*	160	34.04
*Camellia sinensis* L*.*	115	24.46
*Allium sativum* L*.*	100	21.27
*Vitis vinifera* L*.* (water ethanol extract, HPLC identification of the authenticity of the extract, UV measurement of proanthocyanidins (>95%)	40	8.51
Ascorbic acid (PubChem CID: 54670067)	30	6.38
Calcium stearate (PubChem CID: 15324)	10	2.13
Silicon dioxide (PubChem CID: 24261)	8	1.70
DL-alpha-tocopherol (PubChem CID: 2116)	6.6	1.40
Beta-carotene 99% (PubChem CID: 5280489)	0.5	0.11

HPLC: high performance liquid chromatography.
